# Sexuality and mood changes in women with persistent pelvic girdle pain after childbirth: a case-control study

**DOI:** 10.1186/s12905-020-01058-7

**Published:** 2020-09-14

**Authors:** Niklas Rexelius, Anne Lindgren, Thomas Torstensson, Per Kristiansson, Sahruh Turkmen

**Affiliations:** 1 Department of Clinical Sciences, Obstetrics and Gynecology, Umeå University, 90185 Umeå, Sweden; 2 Department of Obstetrics and Gynecology, Sundsvall County Hospital, 851 86 Sundsvall, SE Sweden; 3 Department of Public Health and Caring Sciences, Uppsala University, SE-75622 Uppsala, Sweden

**Keywords:** Pelvic girdle pain, Sexuality, Depression, Women, Postpartum

## Abstract

**Background:**

Pelvic girdle pain is a common problem during pregnancy. For most women, the symptoms cease within the first 3–6 months of giving birth, but in some women the pain persists. In this study we investigate the sexuality and frequency of depressive symptoms in women with persistent pelvic girdle pain after childbirth and in healthy women.

**Methods:**

We conducted a case–control study of women with persistent pelvic girdle pain after childbirth and a control group of healthy women. The frequency of depressive symptoms and sexuality were measured using the self-rating version of the Montgomery–Asberg Depression Rating Scale and the McCoy Female Sexuality Questionnaire.

**Results:**

Forty-six women with persistent pelvic girdle pain and thirty-nine healthy women were enrolled. The frequency of depressive symptoms and the total score on female sexuality did not differ between the groups. However, pain during intercourse was more frequent (*P* < 0.001) in women with persistent pelvic girdle pain and caused them to avoid sexual intercourse frequently (*P* < 0.001). In multiple linear regression a higher frequency of depressive symptoms was reversely correlated with a lower score on female sexuality (*β*_=_ − 0,41, *p* < 0,001 95% CI -0,6 - -0,22) This association remained after adjusting for obstetric variables and individual characteristics.

**Conclusion:**

Depressive symptoms and female sexuality were similar between women with persistent pelvic girdle pain after childbirth and healthy controls. However, pain during intercourse and avoidance of sexual intercourse were more frequent among women with pelvic girdle pain.

## Background

Pelvic girdle pain (PGP) is defined as pain experienced between the posterior iliac crest and the gluteal fold [1], and affects a large proportion of pregnant women with a point prevalence of about 20% [1]. Most women recover within 3–6 months postpartum, but 16–31% suffer persistent pain [2, 3]. Women with more-severe pain during pregnancy are more likely to have pain after they have given birth [4].

Pelvic pain has a broader definition that includes all types of pain in the pelvis, regardless of its etiology, and chronic pelvic pain in women is defined as persistent, noncyclic pain perceived to occur in structures related to the pelvis and lasts for more than 6 months [5]. Women with chronic pelvic pain are more likely to report anxiety and depression, and/or other health conditions [6]. Pelvic girdle pain has a major impact on several aspects of life and women with persistent PGP perceive their health to be worse than do women with recurrent or no pain [7]. It has been suggested that experiencing emotional distress during pregnancy is associated with PGP that persists after delivery [8]. Gutke et al., using the Edinburgh Postnatal Depression Scale (EPDS), reported a higher prevalence of postpartum depressive symptoms in women with lumbar pain and PGP 3 months after delivery than in women without lumbopelvic pain [9]. In an 11-year follow-up study, Elden et al. reported that women with persistent PGP had higher levels of anxiety and depression, reduced ability to perform daily activities, and less working hours per week [10].

The impact of persistent PGP on female sexuality has rarely been studied. Sexual dysfunction is common in women with chronic pain in general, and Verit et al. reported that the prevalence of sexual dysfunction in women with chronic pelvic pain is 67.8%, compared with only 32.3% in a control group of healthy women [11]. Mogren et al. reported that women are less satisfied with their sex lives during pregnancy than before pregnancy, regardless of pain, but are more likely to be dissatisfied if they suffer lumbar or pelvic pain [12]. In a follow-up study, the same authors found that the sexual satisfaction of women with lower back pain and PGP was not restored to prepregnancy levels 6 months after pregnancy [13].

The pathophysiology behind PGP is likely to differ from other types of chronic pelvic and may have different effects on sexuality. Chronic pelvic pain is also associated with depression [6, 14] and depression can affect sexual function. In this study, we compared the sexuality and frequency of depressive symptoms in women with persistent PGP after childbirth and a healthy control group.

## Methods

A case–control study was conducted among women with postpartum PGP and healthy controls. The women were recruited from maternity units in Västernorrland County, Sweden. Women with PGP 3–12 months after childbirth were invited by phone or letter to participate in the study. The control group included healthy women who had undergone delivery in the same period and were recruited separately by telephone or directly though their maternity unit midwife. The background data on the patient and control groups were collected by searching the electronic medical record database for prenatal care and childbirth, Obstetrix (Cerner Corporation, Stockholm, Sweden). All women received written and verbal information about the study from a researcher or research nurse and signed an informed consent form at the time of enrolment.

Women aged ≥18 years and had given birth between July 2015 and July 2018 were eligible. All women who reported PGP with onset during preceding pregnancy were assessed with a general gynecological examination and pain provocation tests of the lower back and pelvis, to ensure that they had PGP. The inclusion criteria in the PGP group were: 1) ongoing sacral pain with onset during the preceding pregnancy; 2) pain intensity in the past week of ≥40 mm on a 100 mm visual analogue scale; 3) at least one positive pain provocation test of either Posterior Pelvic Pain Provocation (P4) test, Menell’s test or Patrick’s Faber test; 4) provoked pain by gentle pressure on the ischial spine ipsilaterally to reported pain on at least one side. In the control group, women who declared that they were pain free were considered healthy and were not examined clinically.

The exclusion criteria were: 1) ongoing pregnancy; 2) nerve root affection in the lumbo-sacral spine; 3) previous fracture or surgery on any pelvic or lumbar bone; 4) inflammatory disease with pelvic bone and/or spinal manifestation; 5) known endometriosis, gynecological cancer or other ongoing malign disease; 6) corticosteroid treatment during the past 6 months; 7) incapacity to participate in an examination or complete the questionnaires.

Demographic information was collected from the electronic medical record database, Obstetrix (Cerner Corporation). This included: parity, time since delivery, mode of delivery, anal sphincter repair, age, body mass index (BMI), smoking habit, medication, and ongoing illnesses during pregnancy.

All of the participants were asked to answer two questionnaires, the self-rating version of the Montgomery–Asberg Depression Rating Scale (MADRS-S) and the nine-item McCoy Female Sexuality Questionnaire (MFSQ) (with permission from Mapi Research Trust, Lyon, France, https://eprovide.mapi-trust.org/), with three extra questions specific for this version of the Swedish translation [15].

The MFSQ measures different aspects of the sex life, which are answered on a 7-point Likert scale [15]. MFSQ has been used in a variety of different settings and multiple languages including Swedish. It was initially validated in post-menopausal women but has shown reliability and validity in university women as well [15]. The Swedish version has been used in studies on oral anticontraceptive [16] and polycystic ovarian syndrome [17]. The original MFSQ contains 19 items, but several studies have used shorter versions, in which the number of items varies from nine to 17. The most frequently used short version contains nine items. We found no argument against the selection of the shorter versions. Our version of the Swedish translation includes three additional questions (items 10–12), which are scored on a five-point Likert scale. We decided to keep them because they cover items relevant to our study. However, to ensure the comparability of our study with other studies that used the nine-item questionnaire, we did not include these three items in the total MFSQ score. The nine questions concerned: 1) satisfaction with the frequency of sexual activity; 2) level of sexual interest; 3) enjoyment of sexual activity; 4) excitement/arousal during sexual activity; 5) frequency of orgasm; 6) insufficient lubrication; 7) painful sexual intercourse; 8) satisfaction with partner as lover; and 9) satisfaction with partner as friend. The three additional questions concerned: 10) frequency of sexual intercourse; 11) frequency of masturbation; and 12) avoidance of sexual intercourse due to pain. Items 6, 7, and 12 were scored reversely so that a high value always represented better sexual function. Both the individual scores for each item and the combined score for items 1–9 were analyzed.

The presence of depressive symptoms was assessed with MADRS-S [18]. MADRS-S is a nine-item diagnostic questionnaire used to measure the severity of depressive episodes in patients with mood disorders. A higher MADRS-S score indicates more-severe depression, and each item is scored from 0 to 6. The overall score ranges from 0 to 54. The questionnaire includes questions on the following symptoms: 1) mood; 2) feelings of unease; 3) sleep; 4) appetite; 5) ability to concentrate; 6) initiative; 7) emotional involvement; 8) pessimism; and 9) zest for life. The questionnaire is not sufficient for a diagnosis but can be used to measure severity of symptoms with good concordance with expert rates scales [19] A Score of 0–12 has been suggested as normal with a sore of ≥13 being suggestive of varying degrees of depression [19]. Due to the limitations of applying a cut-off value we have analyzed the groups using both their total score and into women scoring 0–12 and those scoring ≥13.

### Statistical analysis

SPSS version 25 (IBM Corp., Armonk, NY, USA) was used for data analyses. Descriptive statistics were used to present the data, which are divided into categorical, ordinal, and continuous variables. The Shapiro–Wilk test showed that the data were not normally distributed. Continuous variables were evaluated with the Mann–Whitney *U* test, and categorical and ordinal variables were evaluated with the χ^2^ test. The correlation analysis was performed with Spearman’s rho coefficient. Descriptive statistics are presented as medians (interquartile ranges). Multiple linear regression was performed for MFSQ, combined score item 1–9 as dependent variable and adjusted for potential confounders using: MADRS-S total score, time since birth (days), maternal age (years), BMI (kg/m^2^), parity (primi−/multipara), smoking (yes/no), use of hormonal contraception (yes/no), anal sphincter repair (yes/no) and mode of delivery (vaginal delivery yes/no) as independent variables.

#### Power analysis

According to an earlier study, the prevalence of sexual dysfunction was 67.8% in women with pelvic pain and 32.2% in the general population [11]. Sample size was calculated by comparing two proportions.

To detect a statistically significant difference between women with and without PGP in total score on MFSQ we required at least 39 patients in each group When *β* = 0,1 and *α* = 0,05.

## Results

**Fig. 1:**
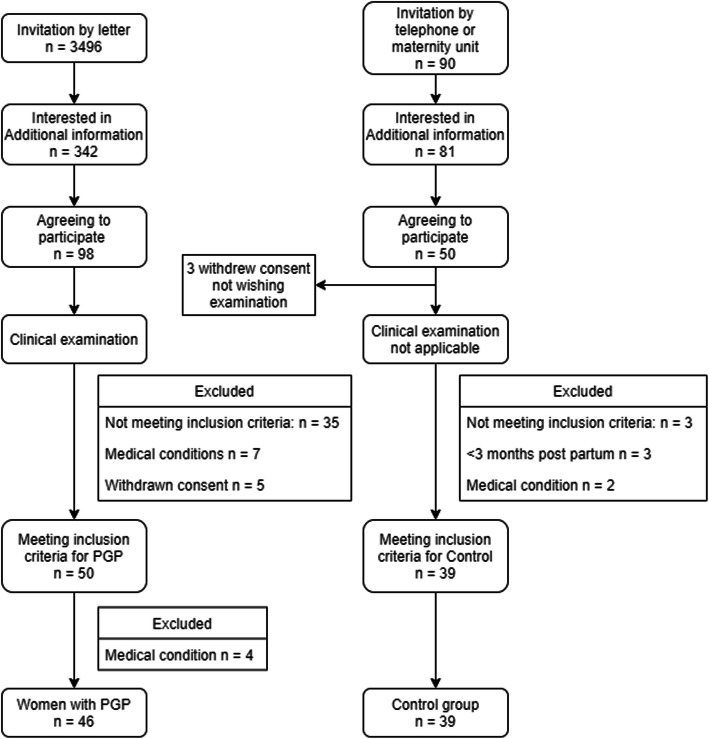
Study flow chart

A total of 100 women were included in the study. The PGP group comprised 53 women and the control group comprised 47 women. After inclusion and answering questionnaires seven women were excluded from the PGP group (three refused or were unable to participate in the examination and four had ongoing medical conditions) and eight women were excluded from the control group (three women because the enrolment time was < 3 months after they had given birth; two suffered other medical conditions; three suffered pain with other causes) (see flow chart, Fig. 1).

The baseline characteristics of both groups are shown in Table 1. No differences between the groups were observed in age, time after delivery, BMI, parity (primi- or multipara), mode of delivery, or the number of perineal or sphincter injuries. In the group of women with PGP, five women reported cigarette smoking, whereas no-one in the control group smoked (*P* = 0.03). Six of the women with PGP used a hormone-based intrauterine device (IUD), as did 12 women in the control group (see Table 1).

**Table 1:** Baseline characteristics and obstetric data

	Patients		Controls		*P* value
	**Median**	**IQR**	**Median**	**IQR**	
**Age (years)**	30.0	8.5	31	5.5	0.553
**Time since delivery (days)**	214	47.5	244	190	0.812
**Body mass index (kg/m**^**2**^**)**	25.1	7.8	24.6	3.9	0.584
	OWLS **n**	OWLS **%**	OWLS **n**	OWLS **%**	
**Parity**					0.504
Primipara	21	45.7	15	38.5	
Multipara	25	54.3	24	61.5	
**Mode of delivery**					0.704
Unassisted	36	78.3	33	84.6	
Vacuum extraction	1	2.2	1	2.6	
Cesarean section	9	19.6	5	12.8	
**Perineal injuries**					0.391
Grades 0–2	43	93.5	38	97.4	
Grades 3–4	3	6.5	1	2.6	
**Smoking during pregnancy**					**0.034**
No	41	89.1	39	100	
Yes	5	10.9	0	0	
**Use of contraception**					0.167
No contraception	30	65.2	20	51.3	
Combined method (pills or patch)	4	8.7	4	10.3	
Gestagen only (pills, implants)	3	6.5	0	0.0	
Hormonal IUD	6	13	12	30.8	
Copper IUD	3	6.5	3	7.7	

*IQR* Interquartile range, *IUD* Intrauterine device

The results of MFSQ were analyzed according to both the sum of items 1–9 and the score for each question. As a main outcome, the total score of item 1–9 there were no significant differences between the groups. There were significant differences between the two groups for items 7 (pain during sexual intercourse) and 12 (avoidance of sexual intercourse due to pain). The women with PGP experienced painful sexual intercourse more frequently and avoided sexual intercourse due to pain more frequently than the control group (Table 2).

**Table 2:** McCoy Female Sexuality Questionnaire and Montgomery–Asberg Depression Rating Scale scores

	**Patients**		**Controls**		***P*** **value**
	Median	IQR	Median	IQR	
**McCoy Female Sexuality Questionnaire**					
Satisfaction with the frequency of sexual activity	5	3	5	3	0.879
Level of sexual interest	3	1	3	1	0.963
Enjoyment of sexual activity	6	3	6	1.5	0.153
Excitement/arousal during sexual activity	7	3	7	1	0.191
Frequency of orgasm	6	3	6	2.5	0.659
Insufficient lubrication	6	3	6	3	0.420
Painful sexual intercourse	5	4	6	1	0.001*
Satisfaction with partner as lover	7	1	7	1.5	0.285
Satisfaction with partner as friend	7	1	7	1	0.868
Frequency of intercourse	3	1	3	1	0.365
Frequency of masturbation	1	1	1	0	0.094
Avoidance of sexual intercourse due to pain.	4	2	5	1	0.000*
Combined score for items 1–9	49	8	52	9	0.100
**Montgomery–Asberg Depression Rating Scale**					
**Total score (0–54)**	6	10	4	12.5	0.138
	n	%	n	%	0.827
score 0–12	34	73.9	28	71.8	
score ≥ 13	12	26.1	11	28.2	

*IQR* Interquartile range

No difference was detected in the total MADRS-S scores of the two groups. The cut-off value for the MADRAS-S score that indicated a need for further investigation was 13. There was no difference in the number of women with MADRS-S scores ≥13 between the two groups (Table 2).

As secondary outcomes: The correlation between the MADRS-S and MFSQ results within each study group was analyzed using Spearman’s rho coefficient. In the PGP group, higher MADRS-S scores correlated negatively with lower satisfaction with the frequency of sexual activity (*r* = − 0.332, *P* < 0.05), the partner as lover (*r* = − 0.309, *P* < 0.05), and sum of the MFSQ scores on questions 1–9 (*r* = − 0.352, *P* < 0.05). There was also a correlation between the time elapsed since birth and the avoidance of sexual intercourse due to pain (*r* = − 0.334, *P* < 0.05), indicating that fewer women avoided intercourse with increasing time since birth.

In the control group, a high MADRS-S score correlated with lower satisfaction and with the frequency of sexual activity (*r* = − 0.347, *P* < 0.05), lower enjoyment of sexual activity (*r* = − 0.390, *P* < 0.05), lower frequency of orgasm (*r* = − 0.539, *P* < 0.01), lower satisfaction with the partner as lover (*r* = − 0.460, *P* < 0.01), lower satisfaction with the partner as friend (*r* = − 0.543, *P* < 0.01), and lower total MFSQ score on questions 1–9 (*r* = − 0.498, *P* < 0.01).

Multiple linear regression analysis shows an association between MFSQ (as dependent variable) and MADRS-S (as independent variable) (*β* = − 0,41, *p* < 0,001 95% CI -0,6 - -0,22) when adjusting for parity, time since birth, age, BMI, use of hormonal contraception, anal sphincter repair and mode of delivery. The same analysis was done for the MFSQ item 7 (pain during intercourse) showing no significant association with MADRS-S.

## Discussion

The frequency of depressive symptoms and the level of female sexual function were similar between women with persistent PGP and women in the control group. In the women with persistent PGP after childbirth, pain was experienced more frequently during sexual intercourse and they also more frequently avoided intercourse due to pain. This suggests that pelvic pain affects the sexual function of women with PGP.

Pelvic girdle pain may affect several aspects of women’s lives, including their sex lives during pregnancy [20] and in the postpartum period [2]. This is consistent with our finding that women with PGP avoid sexual intercourse more frequently because of pain. In the present study, quite intense provoked pain on anatomical landmarks within the small pelvis was an inclusion criterion for all the women with PGP. This could explain the increased pain intensity during penetrative sex reported by women with PGP.

To the best of our knowledge, this is the first detailed controlled study using the MFSQ to assess the sexuality of women with persistent postpartum PGP. In an earlier study, Mogren et al. [12]. used a simple questionnaire with yes/no answers to investigate whether women with PGP were satisfied with their sex lives. They concluded that women were less satisfied with their sex lives during pregnancy and that women with higher pain scores were more likely to be dissatisfied. In a 6-month follow-up study, they found that the women’s satisfaction had not returned to prepregnancy levels, but that it no longer differed between women with different intensity of pain during their pregnancy [13]. A 12-year follow-up study of the same group of women showed that most women were satisfied with their sex lives [21]. Their postpregnancy findings are consistent with how most women rate their sexual interest and activity after childbirth [22]. Tenfelde et al. studied women with musculoskeletal pain (the majority having PGP and pelvic floor myofascial pain) using Female sexuality function index subscales. Women with pain reported lower satisfaction with their sex lives and a greater degree of pain or discomfort associated with vaginal penetration, but had no changes in desire, arousal and orgasm [23]. Their findings of increased discomfort and pain during intercourse for women with PGP are consistent with our results.

Chronic pelvic pain includes all types of pelvic pain that persist for > 6 months, and is associated with a higher level of vaginismus, sexual avoidance, nonsensuality, and sexual dissatisfaction. Both anxiety and depression occur more frequently among women with chronic pelvic pain [14]. Verit et al. [11] concluded that sexual pain disorder was the commonest sexual dysfunction in their study population (women with chronic pelvic pain), and a longer period of pain was associated with higher levels of sexual dysfunction. We detected no correlation between the duration of pain and the MFSQ score in our study. However, in the study by Verit et al., the period of pain among the patients was longer than in our study. It is possible that pain causes more extensive sexual problems if it persists for a long time. In contrast, another study of sexuality in patients of both sexes with any type of chronic pain (predominantly musculoskeletal pain in the lower back, lumbar spine, sacrum, or coccyx) suggested that a shorter period of pain correlated with greater sexual dysfunction and less use of coping strategies to overcome that pain [24]. The authors explained these results by proposing that when patients learn to live with their pain, they report less sexual dysfunction.

Pelvic girdle pain is complex and multifactorial, with no obvious etiology. However, some common factors range from peripheral or central nervous system involvement, altered laxity/stiffness of the muscles or tendinous or ligamentous structures, and ‘maladaptive’ body mechanics [25]. Although it has been suggested that the development of PGP is associated with high levels of ovarian and placental hormones during pregnancy, the evidence is inconsistent [26, 27]. Different types of pain have different pathophysiological mechanisms, with different short- and long-term effects. It is unclear if PGP behaves in the same way as other musculoskeletal pains or chronic pelvic pain.

It have been shown that women with musculoskeletal pain including PGP are more likely to have pelvic floor and levator ani tenderness, but no difference in muscle strength [23]. Increased tenderness of deep pelvic flood muscles with maintained muscle functions has previously been shown in women with PGP during pregnancy [28]. Using a combination of palpation, manometry and 3D ultrasound in women with PGP more than 6 months after birth, Stuge [29] found no differences in voluntary pelvic floor muscle function but levator hiatus was smaller and there was a tendency for a higher resting pressure. These finding might indicate an increased muscle activity to protect from their PGP. An increased muscle activity could lead to muscle tenderness and pain during vaginal intercourse.

It has been suggested that painful intercourse and pain-related psychosocial factors increase the pain experience, but it is unclear how these factors affect one another. Women with pelvic pain may perceive stimuli as more painful than pain-free women when the magnitude of the stimulus is the same [30]. A study of women with chronic pelvic pain found that these patients may show pain-related psychological behavior (including catastrophizing) and hypervigilance during intercourse. It has been demonstrated that women with vulvodynia report hypervigilance to pain during intercourse, suggesting that increased attention to the threat of a painful stimulus during intercourse may interfere with and change the experience of intercourse [31]. However, some studies have offered physically based explanations [32].

The prevalence of depressive symptoms has been studied with different instruments, but the one used most frequently both during and after pregnancy is EPDS. The prevalence of depression in a Swedish population at 6 months postpartum was reported to be 13% on EPDS, when a cut-off of > 10 was used [33]. In our study population, the prevalence of depressive symptoms was higher than expected in both groups. Using a cut-off value of ≥13 for MADRS-S, we detected depression in 26.1% of women with PGP and in 28.2% of women in the control group. Studies have shown a correlation between the MADRS and EPDS [34]. MADRS is rated by a physician and MADRS-S is the self-rated version, and moderate to good agreement between the two scales has been demonstrated [35]. We have found no studies that compared the MADRS-S and EDPS instruments. It is possible that the use of MADRS-S contributed to the higher rate of reported depressive symptoms in our study.

A systematic review recently suggested that pain has negative emotional and psychological effects on patients with pregnancy-related PGP. Women reported feelings of frustration and guilt and were upset because they were unable to carry out their normal roles, and PGP may also have affected the women’s sense of identity and ability to care for their children [36]. A large Norwegian cohort study showed that the risk of persistent PGP after delivery is increased in women who report higher levels of emotional distress during pregnancy, and this remained true after adjustment for pain severity [8]. Similar results were obtained in a study from the Netherlands [37]. In contrast, another Norwegian cohort study found no significant correlation between the emotional distress measured 6–16 weeks after delivery and recovery from PGP at 12 months after delivery [38].

Few studies have used a validated instrument to detect depression in women with PGP. Using EPDS, Gutke et al. [9], suggested that women with lumbopelvic pain are three times more likely to suffer postpartum depressive symptoms than those without pain, and after subgrouping for PGP, the differences were significant for a cut-off value of > 10 (but not for a cut-off of > 13). Similar results were reported in an Australian study of pregnant women, in which lower back pain was associated with an increased risk of depression [39], but that study did not differentiate between lower back pain and PGP. In contrast, in our study, the total MADRS-S score and the number of women scoring ≥13 did not differ between the two groups. Because the association with depressive symptoms was stronger for back pain than for PGP in the study by Gukte et al. [9] and the location of pain was not specified in the Australian study [40], back pain may have a greater effect on psychological wellbeing than PGP. Elden et al. [10] observed higher levels of depressive symptoms and anxiety in women with PGP about 11 years after they had given birth. Therefore, we speculate that a longer period of PGP is required to affect psychological wellbeing than is required for other types of pain.

In women, chronic pelvic pain, regardless of the underlying cause, is correlated with depression [6, 14]. It has been suggested that among women with PGP, the patient’s belief in an early-postpartum improvement in their condition is positive factor, reducing her functional disability and pain in the first year [38]. In other words, if a patient believes that she will become healthy, the pain may affect her mood less strongly during that period.

Associations between depression and sexuality have been demonstrated in both the general population [41] and in women during the first year after childbirth [39, 42]. The correlation we observed between MFSQ and MADRS-S is consistent with those study results.

The number of women who smoked was significantly larger in the group with PGP than in the control groups. European guidelines (2007) state that smoking is not a risk factor for PGP [1], and this has been supported by studies of women with postpartum PGP, [43] although other studies have disagreed [7]. We identified no relationship between smoking and the scores for sexuality or depression.

One of the strengths of this study is that all the women with PGP underwent clinical examinations to confirm the diagnosis. We also used a valid instrument to assess sexuality and as far as we know, MFSQ has not been used previously in a similar population. The study has several limitations which affects the generalizability. We recruited the control group separately from the women with PGP and did not match them to the women with PGP. With limited time and resources, we decided to not examine the women that reported that they are pain free. We cannot be certain what factors prompted women to participate but our analysis of baseline characteristics shows now significant differences between the group except from smoking habits. Since, there was no prior studies on PGP, power calculations in this study was based on sexual dysfunction among women with chronic pelvic pain and not PGP specifically. Given that the mechanism of pain may be different in chronic pelvic pain and PGP, it may have caused the study to be underpowered. On the other side, the study sample was small, which might increase the risk of type 2 error.

## Conclusions

Although women with persistent PGP after childbirth experience more pain during sexual intercourse, their sexuality and frequency of depression did not differ from those of healthy women. The relationship between sexuality and depressive symptoms is important and should be considered when planning treatments for women with persistent PGP. Increasing our knowledge of this health condition will allow the choice of more appropriate treatments for women with PGP.

## Data Availability

The data are available upon reasonable request from the corresponding author.

## Abbreviations

PGP: Pelvic girdle pain
MADRS-S: Montgomery–Asberg Depression Rating Scale, self-rating version
MFSQ: McCoy Female Sexuality Questionnaire
EPDS: Edinburgh Postnatal Depression Scale
IQR: Interquartile range
IUD: Intrauterine device
BMI: Body mass index
